# Knowledge, attitudes and practices of livestock and aquaculture producers regarding antimicrobial use and resistance in Vietnam

**DOI:** 10.1371/journal.pone.0223115

**Published:** 2019-09-25

**Authors:** Phuc Pham-Duc, Meghan A. Cook, Hanh Cong-Hong, Hang Nguyen-Thuy, Pawin Padungtod, Hien Nguyen-Thi, Sinh Dang-Xuan

**Affiliations:** 1 Center for Public Health and Ecosystem Research, Hanoi University of Public Health, Hanoi, Vietnam; 2 Institute of Environment and Sustainable Development, Vietnam Union of Science and Technology Associations, Hanoi, Vietnam; 3 Institute of Materials Science, Vietnam Academy of Science and Technology, Hanoi, Vietnam; 4 Food and Agriculture Organization of the United Nations, Country Office for Vietnam, Hanoi, Vietnam; 5 International Livestock Research Institute (ILRI), Hanoi, Vietnam; University of Campania, ITALY

## Abstract

The use of antibiotics in livestock production is considered a major driver of antibiotic resistance on a global scale. In Vietnam, small- and medium-scale livestock producers dominate the domestic market and regulatory pushes have done little to decrease antibiotic use. In order to inform future policy directions, this study aims to explore knowledge, attitudes, and practices amongst livestock producers to identify their perspectives on antibiotic use and resistance. A total of 392 small- and medium-scale producers specialized in pig, poultry and aquaculture production participated in the study. The results showed that the primary reason for antibiotic use reported by producers was for the treatment of infections (69%). However, prophylactic use was also evident, with farmers reporting other reasons for antibiotic use such as “animals display abnormal symptoms or behaviour” (55%), the “weather is about to change” (25%), or “animals on neighboring farms fall ill” (27%). Only one-fifth of producers demonstrated favorable attitudes towards antibiotic use and preventing antibiotic resistance. Moreover, administering antibiotics remained the preferred countermeasure directly applied by farmers at the first indication of disease (17%), compared to enacting hygiene (10%) or quarantine (5%) measures. The results showed divergent trends amongst producers, with pig producers demonstrating higher levels of knowledge, more favorable attitudes, and higher self-reported utilization of good practice. Better knowledge, attitudes, and practices were also associated with producers who engaged in efforts to explore information on antibiotic use and resistance, which improved incrementally with the number of sources consulted and hours invested. However, there were some areas where increased knowledge or more favorable attitude scores did not translate into better practices. For instance, producers with higher levels of formal education performed significantly better than those with lower education in terms of knowledge and attitude, though both groups reported similar practices. The findings of this study may support future interventions to prevent both antibiotic misuse and the development of antimicrobial resistance.

## Introduction

Antimicrobial use (AMU) in agriculture is considered a major driver of antimicrobial resistance (AMR) [1] and can be to detrimental human health. For species of microorganisms that colonize domestic animals, exposure to antimicrobials acts as a selective evolutionary pressure, facilitating the development of biological resistance mechanisms [2, 3]. As many of the antibiotics used in agriculture are of clinical significance to human medicine, the capacity to treat human infections is being reduced as drug potency becomes progressively diminished [3–5]. In addition, use of antibiotics without veterinary supervision can result in inappropriate drug dosage, which when coupled with insufficient withdrawal periods before slaughter, can result in the final meat products containing antibiotic residues [6, 7]. Above certain levels, these residues can be toxic, thus maximum residue limits for meat products have been set in order to reduce associated health risks [6]. However, even at lower levels which may not confer clinical toxicity, long-term exposure to residues in meat may be involved in the development of chronic diseases and alter compositions of the human microbiota [6].

Governments and international organizations have begun to monitor AMR and regulate the use of antimicrobials, particularly antibiotics. However, current efforts to improve stewardship and monitor AMR are disproportionately leveled at human medicine [8, 9]. Data around use and resistance in agriculture, where a significant portion of the world’s antimicrobials are directed, is difficult to ascertain [4, 10]. Reliable indicators from low- and middle-income countries are particularly scarce, though it is anticipated that growing populations in Africa and Asia will drive increases in antimicrobial use in livestock going into the future [4, 11]. Increased AMU is, in part, due to increasing meat consumption and intensification of livestock farming [4, 12]. Intensification of livestock increases intraspecies and interspecies contact, which can result in the emergence of novel zoonotic pathogens, with Southeast Asia particularly prone to outbreaks [13]. Accordingly, the World Health Organization (WHO) has called for alternative disease prevention measures including improving animal hygiene and housing, evidence-based husbandry practices, and better use of vaccination [14].

In Vietnam, a large amount of antimicrobial use goes unsupervised by veterinarians, particularly as antibiotics are available over-the-counter [7, 15–17]. Over 45 different antibiotics across livestock and aquaculture in Vietnam have been recorded by WHO [18]. Legal antibiotics are often present in livestock feed sold in Vietnam, sometimes alongside other drugs banned by the World Trade Organization, such as chloramphenicol [19]. To combat AMR and antibiotic residues in meat products, the Vietnamese government has issued a Law on Animal Husbandry to become effective in 2020, which will reinforce efforts to control the use of drugs in animal feed and livestock [20]. However, enforcement of regulation remains difficult given the number of smallholder producers in the market, with one estimate suggesting that at least 80% of pork in Vietnam is supplied by smallholder producers [21].

In order to examine AMU behaviours in Vietnamese agricultural sectors, this study surveyed the knowledge, attitudes and practices (KAP) of livestock and aquaculture producers. This study looks to provide a foundation on which to identify future opportunities for further research and initiatives, with a focus on antimicrobial use and prevention of AMR in farming practices.

## Materials and methods

### Study location

**Fig 1:**
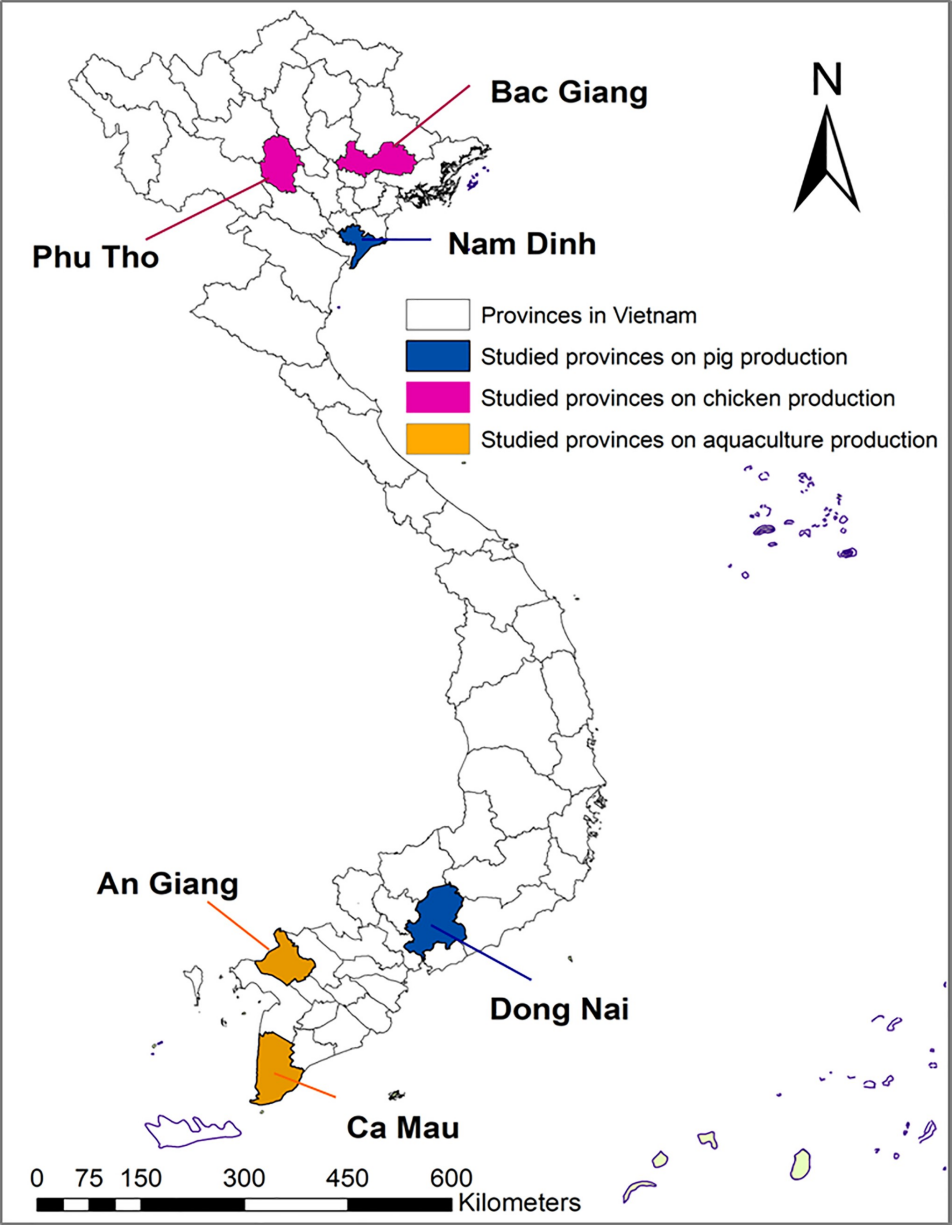
Map of studied provinces by each production type.

Six Vietnamese provinces with high livestock and aquaculture densities were visited in this study. Selected provinces were characterised by high concentrations of a specific type of livestock or aquaculture production, though varied forms of farming existed within each [22]. In Nam Dinh and Dong Nai provinces, the agricultural sector is dominated by small- and medium-scale pig producers who keep less than 500 finishing pigs or 100 sows each. In Bac Giang and Phu Tho provinces, medium-scale chicken producers keeping flocks ranging from 51 to 2000 birds are more common. Lastly, An Giang and Ca Mau provinces were selected for their aquaculture sectors, largely comprised of small- and medium-scale fish and shrimp producers operating on production areas less than two hectares (Fig 1).

### Study design and sample size

This study used a KAP questionnaire to generate cross-sectorial insight, sourcing respondents who participated in different forms of livestock and aquaculture production. Participants were interviewed to elicit their knowledge, attitudes and self-reported practices with regards to antibiotic use (ABU) and resistance.

A single proportion estimation was applied for sample size calculation [23], with a 95% confidence interval, 5% margin of error, and an assumption of that 50% (p = 0.5) of livestock producers use antibiotics in livestock production according to manufacturer recommendations. The minimum sample size required was 384 producers. In the present field survey, a total of 392 producers were interviewed, including 131 pig producers, 127 poultry producers, and 134 aquaculture producers.

### Respondent selection

From each province, two districts with high levels of medium-scale farmers were selected. Within those districts, based on the commune list, further selection processes identified peri-urban and rural commune groups, after excluding urban commune(s), if any. In each commune group, a commune with high level of livestock or aquaculture production was selected. Fifteen to twenty medium-scale producers were randomly selected from the producer list provided by the communal agriculture unit. Given that many producers are small scale farmers, commercial operations were typically conducted on the property of a family household. Researchers requested information from each producer household around which members were involved in livestock production activities. Using the information provided, the person who was judged to have the most responsibility over livestock production in the household was invited to participate in an interview.

### Data collection

Participant responses were gathered using structured quantitative questionnaires. The research team developed questionnaires in English, and then translated the questionnaires into Vietnamese for collection of response information. Another translation back into English was conducted in order to confirm the nuances of the original questions had been adequately captured (copy of survey questionnaires available upon request). Questionnaires were then subjected to a preliminary test in Nam Dinh province with farmers (n = 9), and revised for validation under the supervision of senior researchers. Interviews were conducted in Vietnamese and lasted approximately thirty to forty-five minutes. Responses were then translated into English for further analysis. Major data points captured by the questionnaire included (i) general information of the respondents (e.g. age, gender, education, and main occupation) and their households, (ii) engagement in livestock or aquaculture activities (e.g. type and number of animal, feed type, and rearing methods), (iii) knowledge about AMU and AMR (e.g. familiarity with key terms, knowledge around AMU, definition of AMR, and if any antibiotics are prohibited for use in livestock rearing), (iv) attitudes towards AMU and AMR (e.g. awareness of AMR effects, potential AMU, and attitude towards using antibiotics for disease prevention or growth promotion), (v) practices on AMU and AMR (e.g. first measure undertaken when livestock are sick, when participants would administer antibiotics to animals, and their source of antibiotics), and (vi) exposure to communication and mass media sources (e.g. television channels, time in the day, and duration of viewing). The surveys were conducted between September 2017 and January 2018.

Ethical approval for the study was granted through the Institutional Review Board of Hanoi Public Health University (No. 342/2017/YTCC-HD3). Prospective participants in the study were briefed about how their responses would be used, and informed that they could withdrawal from the study at any time. Those who chose to proceed with the study were asked to review and sign a consent form.

### Data management and analysis

Data obtained from interviews was entered into EpiData (version 3.1, Funen, Denmark) and cross-checked. The data was then extracted into an MS Excel spread sheet for cleaning, processing, and further analysis. Each validated question was independently analyzed with answers assigned a score, either one (correct) or zero (incorrect). To analyse how individual participants performed in each of the knowledge, attitude, and practice categories overall, the sum of each participant’s answers for that section was calculated. Those whose answers were deemed ≥75% correct (or reflected practices that prevent the development of AMR) in a section of the questionnaire were considered to have favorable knowledge, attitudes, or practices, where appropriate. Univariable analysis (Chi square or Fisher exact test, where appropriate) was used to determine factors associated with observed categories. Factors that have p-values ≤ 0.25 were included in the multivariable analysis using Generalized Linear Models (GLM). GLMs with Poisson log linear link function was run in RStudio (glm package) to test the effect of different factors on the outcomes of interest (e.g., knowledge on AB or AMR, attitude and practices on AMU). A backwards selection GLM was used to confirm the final correlations predicted by the univariable analyses. Covariates with a p < 0.05 were included in the final model, while those with p > 0.05 were excluded. Analysis was done using R software (R Core team, 2017) with a two-sided p value ≤ 0.05 considered statistically significant.

## Results

### Participant demographics

Most producers interviewed were male (71%), especially in the aquaculture producer group (97%). Half of participants (50%) were aged between 46 and 60 years old, with another third (32%) aged between 30 and 45 years old. Over half (54%) of participants had achieved secondary (or middle school) levels of education, with another 26% having graduated from high school. Approximately three quarters (73%) of interviewees indicated that livestock production was their primary occupation. The economic status of participants was analyzed in accordance with income thresholds adopted by the Vietnamese government. According to these economic categories, the majority of producers (82%) were considered to be middle-income households or above. For over a third of producers (37%), livestock production contributed to more than 75% of their total income. In addition, 52% of producers had over ten years of experience in livestock production (Table 1).

**Table 1:** Participant socioeconomic demographics.

Item	No. of participants by producer groups, n (%)			
	*Poultry (n = 127)*	*Pig (n = 131)*	*Aquaculture (n = 134)*	*Overall (n = 392)*
**Gender**				
Male	75 (59.1)	73 (55.7)	130 (97.0)	278 (70.9)
Female	52 (40.9)	58 (44.3)	4 (3.0)	114 (29.1)
**Age** (years old)				
<30	2 (1.6)	3 (2.3)	7 (5.2)	12 (3.1)
30–45	36 (28.3)	27 (20.6)	64 (47.8)	127 (32.4)
46–60	71 (55.9)	80 (61.1)	44 (32.8)	195 (49.7)
>60	18 (14.2)	21 (16.0)	19 (14.2)	58 (14.8)
**Education**				
Primary or illiterate	12 (9.5)	22 (16.8)	28 (20.9)	62 (15.8)
Secondary school	70 (55.1)	72 (55.0)	69 (51.5)	211 (53.8)
High school	39 (30.7)	28 (21.3)	33 (24.6)	100 (25.6)
College or higher	6 (4.7)	9 (6.9)	4 (3.0)	19 (4.8)
**Main occupation of interviewers**				
Livestock	93 (73.2)	94 (71.8)	117 (87.3)	304 (77.6)
Other	34 (26.8)	37 (28.2)	17 (12.7)	88 (22.4)
**Years of farming experience** (years)				
≤5	23 (18.1)	25 (19.1)	20 (14.9)	68 (17.3)
6–10	43 (33.9)	36 (27.5)	40 (29.9)	119 (30.4)
11–15	23 (18.1)	25 (19.1)	34 (25.4)	82 (20.9)
≥16	38 (29.9)	45 (34.3)	40 (29.8)	123 (31.4)
**Household size** (No. of members)				
1–2	25 (19.7)	26 (19.8)	3 (2.2)	54 (13.8)
3–4	50 (39.4)	61 (46.6)	63 (47)	174 (44.4)
5–6	40 (31.5)	36 (27.5)	53 (39.6)	129 (32.9)
>6	12 (9.4)	8 (6.1)	15 (11.2)	35 (8.9)
**Household economic status**^a^				
Low income/poverty	28 (22.0)	13 (9.9)	29 (21.6)	70 (17.9)
Middle income and above	99 (78.0)	118 (90.1)	105 (78.4)	322 (82.1)
**Contribution of livestock production to household income** (%)				
<25%	16 (12.6)	25 (19.1)	5 (3.7)	46 (11.7)
25–50%	40 (31.5)	51 (38.9)	16 (11.9)	107 (27.3)
51–75%	34 (26.8)	29 (22.1)	23 (17.2)	86 (21.9)
>75%	33 (26.0)	26 (19.8)	84 (62.7)	143 (36.5)
NA	4 (3.1)	0 (0)	6 (4.5)	10 (2.6)
**Distance from farm to veterinary drug stores** (km)				
≤2.9	115 (90.5)	68 (51.9)	77 (57.5)	260 (66.3)
3–5.9	11 (8.7)	45 (34.3)	42 (31.3)	98 (25.0)
6–9.9	1 (0.8)	12 (9.2)	5 (3.7)	18 (4.6)
≥10	0 (0)	6 (4.6)	10 (7.5)	16 (4.1)

^a^ The economic status of participants was analyzed in accordance with income thresholds adopted by the Vietnamese government.

### Knowledge, attitudes and practices on AMU and AMR in livestock and aquaculture production

#### Knowledge

On average, producers self-reported a higher level of familiarity with antibiotics (80.4%) compared to antimicrobials more broadly (28.6%). In addition, most of the producers (77%) indicated that they had heard of antibiotic resistance. Familiarity with antibiotics and antibiotic resistance was consistent across the different types of producers. Further questions were asked to elucidate whether farmers were merely familiar with terms such as “antibiotic resistance” or had a deeper understanding of resistance as a phenomenon. Farmers were asked to choose from several statements that best captured their understanding of antibiotic resistance (ABR). The most accurate response, “ABR occurs when a strain of bacteria can resist a drug” was selected by approximately 25% of producers. Thirty-two percent (32%) of producers chose the response “ABR happens when antibiotics are no longer effective in treating infection” as the best reflection of their understanding. However, nearly half of all participants (43%) either chose not to answer this question, selected “unsure/do not know”, or knew that ABR was dangerous but did not know how to describe it.

In terms of antibiotics in feed, producers demonstrated differing levels of awareness that “several types of commercial feed contain antibiotics”. Awareness that various feeds may contain antibiotics was relayed by 43% of chicken producers, 32% of pig producers, and 7% of aquaculture producers. The number of producers who did not know whether commercial feeds contain antibiotics was relatively high, at 27% for both pig and chicken producers, and 43% for aquaculture producers. Across all producers, an average of 10% were under the impression that “all types of feed contain antibiotics”. However, the number of producers who thought that “all types of commercial feed contain no antibiotics” was concerningly high, particularly amongst aquaculture producers (44%) as compared to pig (26%) and chicken (19%) producers.

A high proportion of producers did not know of any antibiotics prohibited for use in livestock rearing (34.2%). However, the proportion of aquaculture producers who knew about the prohibition of some antibiotics (57.5%) was significantly higher than that witnessed in pig (27.5%) and chicken (16.5%) producers.

Producers were asked to indicate the sources of communication through which they had intentionally or inadvertently acquired information about antibiotics and animal health. Results indicated that television was the primary source of information in this respect. The second most selected source of information was “conferences/workshops” and the third was radio announcements over community speakers. Producers were also asked to indicate what sources they had intentionally sought out and used to gain information on antibiotics and antibiotic resistance. Television remained the primary source, though the second most sought-after source was direct contact with “people who have the best practices”, and the third most preferred source was community loudspeakers.

#### Attitudes

The vast majority of producers (92%) reported that they were aware of “serious” or “very serious” consequences for the treatment of infections in humans as antibiotics lose their effectiveness. Approximately the same number of producers (93.7%) indicated that loss of effectiveness in the antibiotic used to treat animal disease entailed “serious” or “very serious” consequences.

Seventy-eight percent (78%) of producers “agreed” or “strongly agreed” that antibiotics are necessary for treatment of infectious diseases in humans and animals, while 52% of producers “agreed” or “strongly agreed” that antibiotics are necessary to prevent diseases in humans and animals. The vast majority of producers (95%) reported that proper use of antibiotics could help to reduce the risk of ABR, that it is necessary to stop antibiotic use before slaughter or sale (95%), and that antibiotic use in livestock should be done in consultation with veterinarians (93%). Most producers also “disagreed” (63.8%) or “strongly disagreed” (9.8%) that it is important to use antibiotics to promote growth in livestock production. Producers were relatively divided on whether it is important to use antibiotics for disease prevention in livestock, as 8% “strongly agreed”, 49% “agreed”, 5% were “neutral”, 34% “disagreed”, and 4% “strongly disagreed”. Producers were also polarized over whether antibiotic use is necessary to improve efficiency in livestock production, as 5.8% “strongly agreed”, 36.8% “agreed”, 7.8% were “neutral”, 47.8% “disagreed”, and 1.8% “strongly disagreed”.

Producers “agreed” or “strongly agreed” at relatively consistent levels that ABR has negative impacts on the environment (69.4%), human health (72.7%), livestock production (78%), and household income (81.1%). Some producers did “disagree” that ABR has negative impacts on the environment (14.5%), human health (13.3%), livestock production (11.5%) and household income (9.9%). However, many other producers indicated they were neutral on the issue, with few producers indicating they “strongly disagree” that ABR has negative impacts on the environment (0.8%), human health (0.8%), livestock production (0.3%) and household income (0.3%).

Ninety-three percent (93%) of producers “agreed” or “strongly agreed” that following guidelines and regulations when using antibiotics was an appropriate way to reduce ABR. The majority of producers also “agreed” or “strongly agreed” that livestock need to be properly vaccinated (96%), and that hygiene and biosecurity measures should be applied as part of farm management (95%). Most producers also “agreed” or “strongly agreed” that reducing herd density on farms was another good way to reduce ABR (86%), though this was the least favored biosecurity measure amongst producers.

Ninety-four percent (94%) of producers “agreed” or “strongly agreed” that they needed to be provided with training, assistance, and guidance by veterinarians when using antibiotics. Ninety-two percent (92%) of producers also “agreed” or “strongly agreed” that they need to be provided drug prescriptions and treatment advice by veterinarians when they buy or use antibiotics. Further, the vast majority (94%) of producers “agreed” or “strongly agreed” that drug stores or suppliers should be certified, managed, and frequently monitored by veterinarians. Most producers (97%) also “agreed” or “strong agreed” that a sufficient withdrawal period is required after antibiotic use, before selling livestock destined for slaughter and subsequent human consumption.

#### Practices

**Fig 2:**
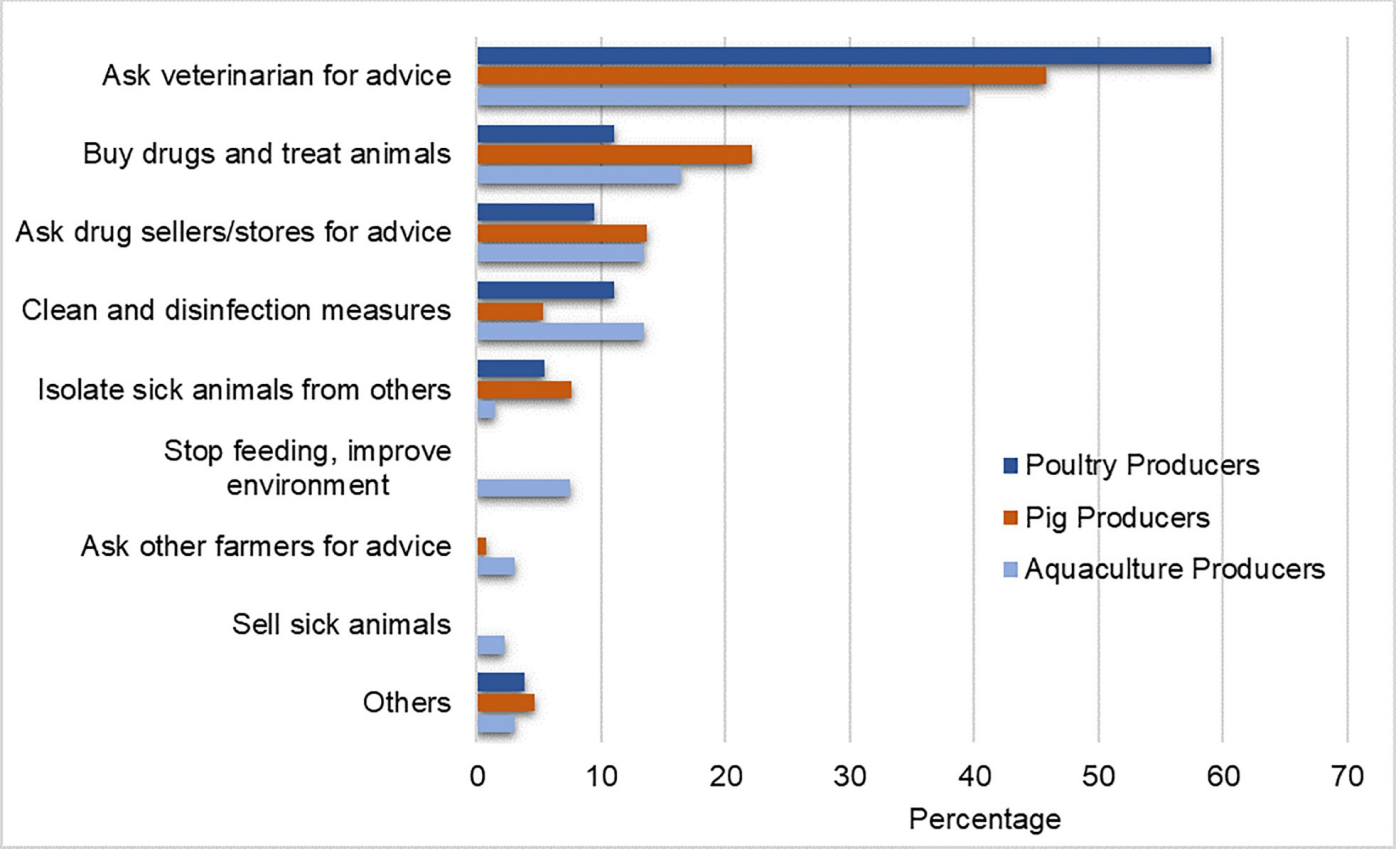
The first response of producers’ (%) when their livestock fall sick.

Producers were asked what their first response would entail if their livestock got sick. Almost half of all producers (48%) said they would first seek the advice of a veterinarian, including 59% of chicken producers, 46% of pig producers, and 40% of aquaculture producers (Fig 2). Pig producers were the most likely to self-administer treatment without veterinary supervision as a first response, with 22% of pig producers indicating that they would decide which drugs to administer and treat their livestock. Thirteen percent (13%) of aquaculture producers, 11% of chicken producers, and 5.5% of pig producers indicated that they would apply cleaning and disinfection measures as a first response when their animals get sick. Measures to isolate sick animals from the rest of the population were also a less popular first response, attracting only 4.8% of all participants. Only a small percentage of aquaculture producers (2%) suggested they would sell their fish or shrimp following signs of illness, the only producer group to do so.

**Fig 3:**
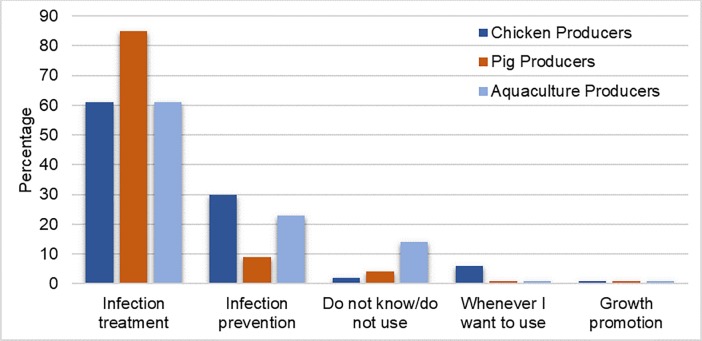
Primary reason for antibiotic use reported by producers.

Most producers in the study self-reported use of antibiotics on their farms. Across all producer groups, the primary reason for antibiotic use was “infection treatment”, equating to 69% of all responses. In descending order of frequency, other producers indicated that their primary reason for antibiotic use was “infection prevention” (20%), “do not know/do not use” (7%), “whenever I want to use” (3%), and “growth promotion” (1%). However, the primary reason cited for antibiotic use showed some minor variation across different sectors. For instance, 85% of pig producers indicated that “infection treatment” was their primary reason for antibiotic use. However, only 61% of chicken and aquaculture producers chose “infection treatment” as their primary reason for antibiotic use. Rather, chicken producers and aquaculture producers were more likely to use antibiotics for “infection prevention” compared to pig farmers (Fig 3).

**Fig 4:**
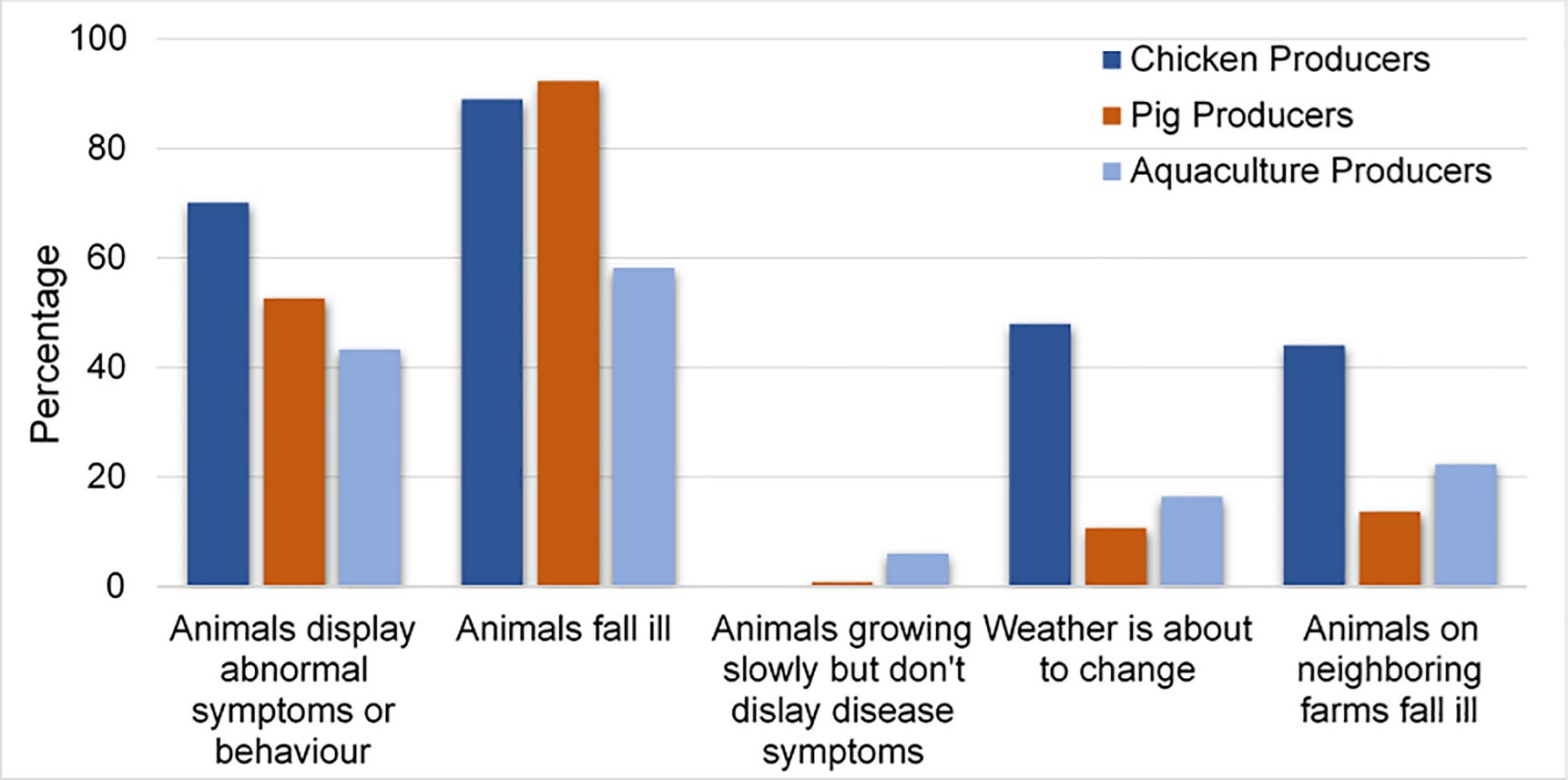
Scenarios in which producers (%) would use antibiotics.

Producers were also asked to indicate all the scenarios in which they would use antibiotics. In response to this question, approximately 1% of pig producers and 6% of aquaculture producers indicated they would use antibiotics if their animals were growing slowly but did not display any symptoms of disease. The remaining scenarios that indicated prophylactic use attracted a higher level of response across all producer groups: “animals display abnormal symptoms or behaviour” (55%), “weather is about to change” (25%), and “animals on neighboring farms fall ill” (27%, Fig 4).

The prophylactic use of antibiotics was explored further through this question, yielding a comparable response rates to both “weather is about to change” and “animals on neighboring farms fall ill” responses. A scenario in which “animals fall ill” remained the top response, with “animals displaying abnormal symptoms or behaviour” being the second most popular answer, though the content of the response was ambiguous regarding the presence of a clinical illness. Chicken producers remained more likely to indicate prophylactic use than aquaculture and pig producers. Here, responses that demonstrated growth promotion effects of antibiotics were also selected at higher levels, indicating that while growth promotion may not be the primary reason for AMU amongst producers, it may still occur, particularly amongst aquaculture producers.

Those producers who indicated that they would use antibiotics when “animals fall ill” (Fig 4) were asked for the main information source that would inform what antibiotics they chose to administer. Veterinarians were the most common source of advice on antibiotics when animals fall ill, chosen by 41% of producers. However, “own experience” came in at a close second, with 35.3% of producers indicating that response. The other major source of advice was a “drug seller”, though drug sellers were more popular among chicken producers (25.7%) as opposed to aquaculture (16.7%) and pig producers (14%). Other primary sources of information selected by a minority of producers included “other producers”, “friends”, and “relatives”.

Eleven percent (11%) of chicken producers, 13% of pig producers, and 9% of aquaculture producers bought antibiotics directly from veterinarians. However, over-the-counter purchases dominated, with drug sellers or drug stores indicated as the site of antibiotic purchases for 89% of chicken producers, 87% of pig producers, and 84.4% of aquaculture producers. In addition, 3.4% of aquaculture producers also indicated that they purchased antibiotics from other farmers, the only producer group to do so.

Most of the producers indicated that they observed withdrawal periods and stopped using antibiotics before selling animals for slaughter, as per manufacturer’s instruction. However, observance of withdrawal periods was self-reported by chicken producers (96.9%) and pig producers (94.7%) at higher rates than aquaculture producers (86.6%). Most producers (82.4%) indicated that they would stop using antibiotics if they expired. However, a small number of aquaculture producers (0.7%) and pig producers (0.8%) indicated that they would continue to use antibiotics if they expired at an increased dosage.

### Factors related to KAP on AMU and AMR in livestock and aquaculture production

Results from a univariable analysis on “knowledge” indicated that 92.5% of producers had insufficient knowledge regarding antibiotics and 57.9% had insufficient knowledge around ABR, according to the parameters of this study. Results demonstrated that education levels were positively correlated with increased levels of knowledge around antibiotics, with producers attaining a high school education or higher found to have better knowledge than those who did not attain a completed high school education (OR = 2.37, 95%CI = 1.44–3.88, Table 2). The differences in knowledge between producers from the provinces of Ca Mau and An Giang, each chosen for inclusion for their high rates of aquaculture production, were significant (OR = 8.61, 95%CI = 2.71–39.9). The province of An Giang was one of the best performing provinces in terms of knowledge on antibiotics and ABR, where Ca Mau was the worst performing province. Producers with an income of between 5 and 10 million Dong per month performed better that higher and lower income groups across both categories (OR = 0.43, 95%CI = 0.22–0.81). Moreover, those who self-reported that they kept records of antibiotic use performed better than those who did not (OR = 0.55, 95%CI = 0.34–0.89). Knowledge also increased with the number of sources producers used to acquire information around antibiotics and ABR (OR = 0.35, 95%CI = 0.13–0.87, Table 2).

**Table 2:** Univariable analysis on the knowledge of interviewed producers.

Variable	N	Knowledge on antibiotic			Knowledge on antibiotic resistance		
		Insufficient (n (%))	OR_crude_	95%CI	Insufficient (n (%))	OR_crude_	95%CI
**Gender**							
Female	114	92 (80.7)			70 (61.4)		
Male	278	212 (76.3)	0.77	0.44–1.31	157 (56.5)	0.82	0.52–1.72
**Education**							
Completed high school or higher	119	79 (66.4)			60 (50.4)		
Secondary school or less	273	225 (82.4)	**2.37**^a^	**1.44–3.88**	167 (61.2)	**1.55**	**1.00–2.39**
**Province**							
An Giang	65	46 (70.8)			35 (53.8)		
Bac Giang	66	48 (72.7)	1.1	0.51–2.38	33 (50.0)	0.86	0.43–1.71
Ca Mau	69	66 (95.7)	**8.61**	**2.71–39.9**	47 (68.1)	1.82	0.90–3.72
Dong Nai	68	48 (70.6)	0.99	0.47–2.11	43 (63.2)	1.47	0.73–2.97
Nam Dinh	63	48 (76.2)	1.32	0.60–2.95	28 (44.4)	0.69	0.34–1.38
Phu Tho	61	48 (78.7)	1.52	0.67–3.50	41 (67.2)	1.75	0.85–3.65
**Household income** (Million VND/month)							
11 million and above	54	43 (79.6)			39 (72.2)		
5–10 million	213	157 (73.7)	0.72	0.33–1.46	112 (52.6)	**0.43**	**0.22–0.81**
Less than 5milion	96	78 (81.3)	1.11	0.47–2.56	65 (67.7)	0.81	0.38–1.68
**Producer group**							
Poultry	127	96 (75.6)			74 (58.3)		
Aquaculture	134	112 (83.6)	0.64	0.98–3.06	82 (61.2)	1.13	0.68–1.86
Pig	131	96 (73.3)	0.89	0.50–1.56	71 (54.2)	0.85	0.52–1.39
**Number of years of keeping pig (chicken or aquaculture)**							
10 years or less	187	149 (79.7)			113 (60.4)		
11 years or more	205	155 (75.6)	0.79	0.49–1.28	114 (55.6)	0.82	0.55–1.23
**Contribution of livestock to household income**							
50% or less	153	116 (75.8)			94 (61.4)		
More than 50%	229	178 (77.7)	1.11	0.68–1.81	125 (54.6)	0.76	0.50–1.15
**Number of diseases reported in a year**							
None	50	39 (78.0)			27 (54.0)		
One	85	67 (78.8)	1.05	0.44–2.45	70 (82.4)	**3.92**	**1.79–8.82**
Two or more	257	198 (77.0)	0.95	0.44–1.93	130 (50.6)	0.87	0.47–1.61
**Keep record of using antibiotics**							
No	249	203 (81.5)			151 (60.6)		
Yes	143	101 (70.6)	**0.55**	**0.34–0.89**	76 (53.1)	0.74	0.49–1.12
**Number of information source types**							
None of any type	41	34 (82.9)			19 (46.3)		
One source type	103	82 (79.6)	0.81	0.29–2.04	54 (52.4)	1.27	0.61–2.66
Two sources types	75	47 (62.7)	**0.35**	**0.13–0.87**	32 (42.7)	0.86	0.40–1.87
More than two source types	83	51 (61.4)	**0.33**	**0.12–0.82**	32 (38.6)	0.73	0.34–1.67
**Information from other persons**							
No	140	103 (73.6)			72 (51.4)		
Yes	162	111 (68.5)	0.78	0.47–1.29	65 (40.1)	**0.63**	**0.40–1.00**

OR, Odd ratio; CI, Confidence interval.

^a^ Values in bold were indicated significantly difference with the reference category.

Overall, 20.4% of producers had favorable attitudes towards antibiotic use while 21.4% of producers had attitudes that were favorable in terms of preventing antibiotic resistance. Higher levels of education, smaller scale farming, keeping livestock penned, keeping records of antibiotic use, and sufficient levels of knowledge were all positively correlated with more favorable attitudes towards ABU and ABR (Table 3). Here, poultry farmers performed worse than their counterparts in pig and aquaculture production, with Phu Tho the worst performing province of all. Favorable attitudes towards ABU and ABR also positively correlated with the number of sources used by producers to acquire knowledge around antibiotics (OR = 0.34, 95%CI = 0.11–0.94), as well as the amount of time spent exploring these sources (OR = 0.29, 95%CI = 0.12–0.63, Table 3).

**Table 3:** Univariable analysis on the attitude of interviewed producers.

Variable	N	Attitude towards antibiotic use			Attitude towards antibiotic resistance		
		Unfavorable attitude (n (%))	OR_crude_	95%CI	Unfavorable attitude (n (%))	OR_crude_	95%CI
**Gender**							
Female	114	92 (80.7)			98 (86.0)		
Male	278	220 (79.1)	0.91	0.52–1.56	210 (75.5)	**0.51**^a^	**0.27–0.90**
**Education**							
Secondary school or higher	119	90 (75.6)			85 (71.4)		
Secondary school or less	273	222 (81.3)	1.4	0.83–2.35	223 (81.7)	**1.78**	**1.07–2.94**
**Province**							
An Giang	65	53 (81.5)			52 (80.0)		
Bac Giang	66	58 (87.9)	1.63	0.62–4.51	55 (83.3)	1.25	0.51–3.11
Ca Mau	69	59 (85.5)	1.33	0.53–3.43	52 (75.4)	0.77	0.33–1.75
Dong Nai	68	54 (79.4)	0.88	0.36–2.09	55 (80.9)	1.06	0.44–2.53
Nam Dinh	63	33 (52.4)	**0.25**	**0.11–0.55**	41 (65.1)	0.47	0.21–1.04
Phu Tho	61	55 (90.2)	2.04	0.73–6.35	53 (86.9)	1.64	0.63–4.51
**Producer group**							
Poultry	127	113 (89.0)			108 (85.0)		
Aquaculture	134	112 (83.6)	0.63	0.30–1.30	104 (77.6)	0.61	0.32–1.15
Pig	131	87 (66.4)	**0.25**	**0.12–0.47**	96 (73.3)	**0.49**	**0.26–0.90**
**Farm scale**							
Medium	205	172 (83.9)			166 (81.0)		
Small	187	140 (74.9)	**0.57**	**0.35–0.94**	142 (75.9)	0.74	0.46–1.21
**Type of animal keeping**							
Both penned and free ranging	68	62 (91.2)			61 (89.7)		
Penned	137	92 (67.1)	**0.20**	**0.07–0.47**	100 (73.0)	**0.32**	**0.12–0.72**
Free ranging	183	154 (84.2)	0.52	0.19–1.25	143 (78.1)	**0.42**	**0.16–0.94**
**Kept records of using antibiotic**							
No	249	207 (83.1)			201 (80.7)		
Yes	143	105 (73.4)	**0.56**	**0.34–0.93**	107 (74.8)	0.71	0.43–1.17
**Number of information source types**							
None of any type	41	34 (82.9)			36 (87.8)		
One source type	103	87 (84.5)	1.13	0.40–2.93	82 (79.6)	0.56	0.17–1.50
Two sources	75	57 (76.0)	0.66	0.23–1.71	53 (70.7)	**0.34**	**0.11–0.94**
More than two source types	83	56 (67.5)	0.44	0.16–1.07	55 (66.3)	**0.28**	**0.09–0.75**
**Duration of exploring information**							
1.0 hour or less	85	76 (89.4)			75 (88.2)		
1.1 to 2.0 hours	108	92 (85.2)	0.69	0.27–1.63	90 (83.3)	0.67	0.28–1.53
2.1 to 4.0 hours	84	67 (79.8)	0.47	0.19–1.12	65 (77.4)	0.46	0.19–1.05
4.1 to 6.0 hours	78	49 (62.8)	**0.2**	**0.08–0.45**	53 (67.9)	**0.29**	**0.12–0.63**
More than 6.0 hours	27	18 (66.7)	**0.24**	**0.08–0.71**	17 (63.0)	**0.23**	**0.08–0.65**
No exploring	10	-	-	-	8 (80.0)	0.51	0.10–4.16
**Sufficient knowledge on AB**							
Yes	88	63 (71.6)			54 (61.4)		
No	304	249 (81.9)	**1.80**	**1.30–3.09**	254 (83.6)	**3.19**	**1.88–5.40**
**Favourable attitude towards AMU**							
Yes	80	-			33 (41.3)		
No	312	-	-	-	275 (88.1)	**10.5**	**6.0–18.6**

OR, Odd ratio; CI, Confidence interval.

^a^ Values in bold were indicated significantly difference with the reference category.

Overall, 59% of livestock producers were calculated to have adequate practices in line with good antibiotic stewardship based on their responses. Unfavorable practices pertaining to antibiotic use were observed at their highest levels amongst aquaculture producers (52.2%, Table 4) who were followed closely by chicken producers (50.4%). When it came to self-reported practices, the proportion of males who reported unfavorable practice was significantly higher than the proportion of females (OR = 1.71, 95%CI = 1.09–2.74). Those producers who caged their livestock demonstrated better practices than those who let their livestock roam freely, even if in combination with some time caged. In addition, the more time producers invested in gaining information on antibiotics, the better the self-reported practices (OR = 0.45, 96%CI = 0.24–0.83). Also, those who kept records of previous antibiotic use reported marginally better practices than those who did not (OR = 0.69, 95%CI = 0.45–1.06). Better AMU practices were associated with sufficient knowledge around antibiotics (OR = 1.85, 95%CI = 1.12–3.12), and favorable attitude scores towards ABU (OR = 4.48, 95%CI = 2.44–8.82, Table 4).

**Table 4:** Univariable analysis on the AMU practice of interview producers.

Variable	Antimicrobial use practices			
	N	Unfavorable practices (n (%))	OR_crude_	95%CI
**Gender**				
Female	114	36 (31.6)		
Male	278	123 (44.2)	**1.71**^a^	**1.09–2.74**
**Education**				
Secondary school or higher	119	50 (42.0)		
Secondary school or less	273	109 (39.9)	0.92	0.59–1.42
**Province**				
An Giang	65	25 (38.5)		
Bac Giang	66	37 (56.1)	**2.03**	**1.01–4.12**
Ca Mau	69	45 (65.2)	**2.97**	**1.48–6.10**
Dong Nai	68	13 (19.1)	**0.38**	**0.17–0.83**
Nam Dinh	63	12 (19.0)	**0.38**	**0.17–0.84**
Phu Tho	61	27 (44.3)	1.27	0.62–2.60
**Producer group**				
Chicken	127	64 (50.4)		
Aquaculture	134	70 (52.2)	1.08	0.66–1.75
Pig	131	25 (19.1)	**0.23**	**0.13–0.41**
**Method of animal keeping**				
Both cage and free	68	34 (50.0)		
Cage	137	29 (21.2)	**0.27**	**0.14–0.51**
Free	183	93 (50.8)	1.03	0.59–1.81
**Number of diseases reported in a year**				
None	50	30 (60.0)		
One	85	26 (30.6)	**0.30**	**0.14–0.61**
Two or more	257	103 (40.1)	**0.45**	**0.24–0.83**
**Keep record of using antibiotics**				
No	249	109 (43.8)		
Yes	143	50 (35.0)	0.69	0.45–1.06
**Duration of exploring information**				
1.0 hour or less	85	43 (50.6)		
1.1 to 2.0 hours	108	44 (40.7)	0.67	0.38–1.20
2.1 to 4.0 hours	84	30 (35.7)	0.54	0.29–1.01
4.1 to 6.0 hours	78	25 (32.1)	**0.46**	**0.24–0.88**
More than 6.0 hours	27	9 (33.3)	0.49	0.19–1.21
No exploring	10	8 (80)	3.67	0.83–27.9
**Sufficient knowledge on AB**				
Yes	88	26 (29.5)		
No	304	133 (43.8)	**1.85**	**1.12–3.12**
**Desirable attitude towards ABU**				
Yes	80	13 (16.3)		
No	312	146 (46.8)	**4.48**	**2.44–8.82**

OR, Odd ratio; CI, Confidence interval.

^a^ Values in bold were indicated significantly difference with the reference category.

Results from a multivariable analysis on knowledge about antibiotics and AMR, attitude towards AMU and AMR, as well as practices around AMU indicated the significant results in the five final models (Table 5).

**Table 5:** Multivariable analysis result.

Final generalized linear models and variable	OR_adjusted_	95%CI	*p*-value
***Model 1*.*KAB*** *(factors relevant to insufficient knowledge on antibiotics)*			
***Education***			
Completed high school or higher	Ref		
Secondary school or less	2.16	**1.24–3.76**^a^	**0.007**
***Province***			
An Giang	Ref		
Bac Giang	1.46	0.63–3.37	0.373
Ca Mau	8.56	**2.25–32.56**	**0.002**
Dong Nai	0.77	0.33–1.76	0.532
Nam Dinh	1.51	0.64–3.56	0.348
Phu Tho	1.25	0.51–3.08	0.630
***Number of information source types***			
None of any types	Ref		
Once source type	0.66	0.25–1.75	0.406
Two source types	0.3	**0.11–0.8**	**0.016**
More than two source types	0.25	**0.09–0.65**	**0.005**
**Model 2. KAMR** *(factors relevant to insufficient knowledge on antibiotic resistance)*			
***Household income*** *(Million*: *11*, *5–10*, *<5)*			
Over 11 mil	Ref		
5–10 mil	0.7	0.35–1.39	0.364
Less than 5 mil	0.37	**0.17–0.79**	**0.004**
***Number of years of keeping pig***			
10 years or less	Ref		
11 years or more	0.68	0.43–1.06	0.089
***Contribution of livestock to household income***			
50% or less	Ref		
More than 50%	1.05	0.96–1.14	0.275
***Number of diseases reported in a year***			
No diseases present at farm last year	Ref		
One disease present at farm last year	6.48	**2.71–15.49**	**<0.001**
Two or more diseases present at farm last year	1.29	0.65–2.55	0.461
**Model 3. AAMU** *(factors relevant to unfavorable attitude on antibiotic use)*			
***Province***			
An Giang	Ref		
Bac Giang	2.59	0.94–7.13	0.066
Ca Mau	1.99	0.76–5.21	0.161
Dong Nai	1.18	0.48–2.89	0.718
Nam Dinh	0.44	0.19–1.03	0.058
Phu Tho	**3.33**	**1.12–9.91**	**0.031**
***Duration of exploring information***			
1.0 hour or less	Ref		
1.1 to 2 hours	0.61	0.25–1.49	0.278
2.1 to 4.0 hours	0.48	0.19–1.18	0.108
4.1 to 6.0 hours	0.2	**0.08–0.49**	**<0.001**
More than 6.0 hours	0.24	**0.08–0.75**	**0.014**
**Model 4. AAMR** *(factors relevant to unfavorable attitude toward antibiotic resistance)*			
***Gender***			
Female	Ref		
Male	0.47	**0.24–0.93**	**0.030**
***Sufficient knowledge on AB***			
No	Ref		
Yes	3.08	**1.68–5.66**	**<0.001**
***Desirable attitude towards ABU***			
No	Ref		
Yes	10.9	**6.03–19.69**	**<0.001**
**Model 5. PAMU** *(factors relevant to unfavourable antimicrobial use practices)*			
***Province***			
An Giang	Ref		
Bac Giang	2.86	**1.32–6.22**	**0.008**
Ca Mau	4.24	**1.91–9.39**	**<0.001**
Dong Nai	0.39	**0.16–0.95**	**0.039**
Nam Dinh	0.66	0.27–1.63	0.371
Phu Tho	1.51	0.64–3.57	0.344
***Number of diseases reported in a year***			
No diseases present at farm last year	Ref		
One disease present at farm last year	0.2	**0.08–0.46**	**<0.001**
Two or more diseases present at farm last year	0.31	**0.14–0.65**	**0.002**
***Duration of exploring information***			
1.0 hour or less	Ref		
1.1 to 2 hours	0.55	0.29–1.05	0.070
2.1 to 4.0 hours	0.49	**0.24–0.98**	**0.043**
4.1 to 6.0 hours	0.45	**0.21–0.96**	**0.039**
More than 6.0 hours	0.41	0.14–1.19	0.101
***Desirable attitude towards ABU***			
No	Ref		
Yes	3.22	**1.61–6.44**	**0.001**

OR, Odd ratio; CI, Confidence interval.

^a^ Values in bold were indicated significantly difference with the reference category.

## Discussion

Vietnamese producers included in this study reported a number of varied reasons for antibiotic use. At first glance these results are favorable, as the majority of producers (69%) indicated their primary reason for antibiotic use was in response to infections, rather than as a prophylactic measure (20%) or for growth promotion side effects (1%). Low utilization of antibiotics for growth promotion effects was supported by a follow-up question, asking producers to indicate different scenarios in which they would use antibiotics more generally. In response to this question, approximately 1% of pig producers and 6% of aquaculture producers indicated they would use antibiotics if their animals were growing slowly but didn’t display any symptoms of disease. However, responses also demonstrated that while prophylactic use may not be the primary reason for utilization of antibiotics, a substantial amount of prophylactic use may occur. The remaining scenarios that indicated prophylactic use attracted a higher level of response, such as “weather is about to change” (25%) and “animals on neighboring farms fall ill” (27%). As such, administration of antibiotics appears to occur for real, imagined, and anticipated infections.

Results demonstrated that acquiring antibiotics was a preferred response to disease outbreak over other disease reduction or prevention measures. When asked what their first response to a potential outbreak would be, 48% of producers indicated that they would seek veterinary attention. A further 17% of producers indicated that they would prefer to buy drugs and treat the animals directly by themselves. Buying drugs to treat the animals directly was reported twice as much amongst pig producers as poultry producers. These technical measures were far more popular than infection control and prevention measures such as cleaning the farm environment (10%) or quarantining sick animals (5%).

Demographic information of the participants may give some context to antibiotic use results. The majority of producers included in this study were aged between 30 and 60 years old (81%) with livestock as their main occupation (78%). For over a third of producers (37%), this income from livestock contributed to over 75% of their household income. Many producers also had an education lower than high school level (71%). With the overall picture this data presents, it is reasonable to suggest that raising livestock may be one of a limited set of employment options for many producers, particularly in a regional area. Moreover, the income generated through livestock rearing may be central for many to staying out of poverty, with 18% of producers in this study classified as poor or in poverty despite income from livestock. These demographics may help to explain that, while producers do show concern around the effects of antibiotics on human and animal health, producers reported slightly higher concern about the impact ABR may have on household income. Given antibiotics are easily available in Vietnam and help facilitate intensive farming, it is perhaps unsurprising that Vietnamese producers utilize antibiotics to guarantee the health of their livestock and ensure their labor is profitable.

In terms of acquiring information on antibiotics and antibiotic resistance, a higher number of sources of information consulted by producers correlated with more favorable knowledge and attitude results, though there are notable discrepancies between comparable knowledge and attitude figures. Producers had incrementally higher levels of knowledge around antibiotics and ABR as the number of sources consulted increased (Table 2). However, sufficient levels of knowledge around ABR were significantly higher than that of antibiotics at each increment. For instance, for producers who consulted no sources of information, only 17% had sufficient knowledge on antibiotic, where 54% had sufficient knowledge on ABR. These results may reflect the information contained within the sources, which may focus on communicating risk associated with ABR and contain less information on antibiotics more broadly. While attitudes towards ABU and ABR also became incrementally more favorable as the number of sources of information increased, there was no discernible discrepancy between attitudes toward ABU and ABR. Given that knowledge around ABR at each increment was significantly higher than that of comparable attitude figures, the results demonstrated that increased knowledge does not necessarily translate into more favorable attitudes towards ABR. The length of time (hours) spent exploring information on antibiotic and ABR, as self-reported by producers, also produced more favorable attitude and practice results. However, as increased levels of knowledge did not necessarily translate into more favorable attitudes and unfavorable attitudes did not necessarily translate into less favorable practices; indicators demonstrated that producers’ self-reported practices may be better than the attitude results would otherwise suggest.

A number of other indicators emerged to be conducive to better knowledge, attitude and practice values. For instance, producers who indicated that they kept records of antibiotic use attained more favorable scores than producers who did not across the knowledge, attitude and practice components of the questionnaire. Producers who reported that they only kept their animals penned without the opportunity to roam also attracted more favorable results in the attitudes and practice components. Further, higher levels of knowledge and favorable attitudes regarding the use of antibiotics correlated with better reported practices when using antibiotics. However, the effect of baseline education generated some divergent results Producers with a lower level of formal education performed worse in the knowledge and attitude components as compared to producers with higher levels of education, though reported comparable practices. In addition, female producers reported better AMU practices than of male producers.

Producers in the study reported some practices which could inform future directions to prevent misuse of antibiotics and development of ABR. However, top-down regulatory approaches to antibiotic sales and use are likely to be constrained funding and logistical challenges. As such, initiatives to reward the responsible prescription and distribution of antibiotics, or promote antibiotic stewardship from the community level, would likely foster more success. Interventions that suit context-specific administration behaviours should be facilitated. However, prophylactic use of antibiotics will continue to occur unless global action is taken to remove antibiotics from animal feed. While the Vietnamese government is taking steps to increase regulation around antibiotic use in agriculture [20], many farmers in this study remained unaware of any antibiotics already prohibited in livestock rearing. Educational campaigns may help improve producer awareness, though the inclusion of antibiotics in feed products undermines meaningful progress towards reducing ABR. Removal of antibiotics from all commercially produced feed is a necessary step going forward, though will be difficult to achieve without sufficient political will or the input of professions and sectors outside of health and pharmaceuticals.

This study has some limitations due to the nature of attaining data on human behaviours through survey tools. The data in this study was collected from participants using a knowledge, attitude and practice (KAP) questionnaire. While the KAP survey tool enables large amounts of data to be captured from a significant number of research participants over a short period, data collected can misrepresent true dispositions and practices. As participants self-report outlooks and previous behaviours, such data may be detrimentally affected by inaccurate recall or confirmation bias, particularly if the subject matter concerns a contentious topic or practice. In addition, KAP survey tools can inadvertently cause participants to offer answers that they anticipate the researcher views as correct or favorable. As such, while much effort has gone into maintaining the accuracy and integrity of the data resulting from this research, the abovementioned factors should be noted.

## Conclusion

Vietnamese producers included in this study reported several varied reasons for antibiotic use, including infection treatment, prophylactic use, and a small amount of use for growth promotion purposes. Results further suggest that in preventing or addressing a disease outbreak, producers preferred technical interventions such as vaccination or obtaining antibiotics, over other measures that alter the farm environment or quarantine sick animals. In addition, understanding demographic information of the participants, such as education, income source, or age, also plays important to influence properly use and attitudes toward antibiotic use and antibiotic resistance. Nevertheless, many producers indicated that they invested time into gathering information on and keep record on antibiotics and ABR, which is positively correlated with better knowledge, attitude and practice results. Overall, pig producers performed the best across three studied producers, with aquaculture producers forming worse than the others, though these results were not always consistent across themes. The antimicrobial use and antibiotic resistance behaviours reported by producers in this study should inform ongoing research and policy changes to prevent antibiotic misuse and the development of antibiotic resistance.

## Supporting information

S1 File (XLSX)

S2 File (XLSX)
